# Correction: Orthogonal Invariant Sets of the Diffusion Tensor and the Development of a Curvilinear Set Suitable for Low-Anisotropy Tissues

**DOI:** 10.1371/annotation/7c0cda94-b886-4654-a7e9-ec52861d1784

**Published:** 2014-01-23

**Authors:** Robin A. Damion, Aleksandra Radjenovic, Eileen Ingham, Zhongmin Jin, Michael E. Ries

Errors were introduced into Equation 2 during production. The correct version of Equation 2 can be found here: http://plosone.org/corrections/pone.0047915.e002.cn.tif

